# Pharmacotherapy Update and Review for Family Medicine Residents Using Jeopardy-Style Game

**DOI:** 10.15766/mep_2374-8265.10941

**Published:** 2020-07-30

**Authors:** Giselle Ellis

**Affiliations:** 1 Assistant Professor, Department of Family Medicine, Wright State University Boonshoft School of Medicine

**Keywords:** Pharmacotherapy, Medications, Guidelines, Family Medicine, Games, Pharmacist, Pharmacology and Toxicology

## Abstract

**Introduction:**

Pharmacotherapy is an important, required aspect of family medicine residency training. *MedEdPORTAL* has very limited pharmacotherapy content that is targeted to a graduate medical education audience.

**Methods:**

I implemented a Jeopardy-style game during a 1-hour didactic session to actively engage the family medicine residents. The game focused on reinforcing guidelines and teaching new medications. I created a session-specific evaluation tool to assess the residents' enjoyment of and learning from the activity.

**Results:**

Twenty-six family medicine residents participated in the session, working in groups of three or four. I evaluated the session using the session-specific evaluation tool and a standard didactics evaluation. Twenty-three of 26 residents completed the session-specific evaluation; all 26 completed the standard evaluation. All the residents agreed or strongly agreed that the session was enjoyable, an opportunity for learning, and something they would look forward to in the future. All the residents also agreed that the information presented applied to clinical practice. Comments primarily focused on the difficulty of the questions and the enjoyment of the session.

**Discussion:**

Based upon the results of the evaluations and comments, the residents felt the session was a valuable opportunity for learning. The session could be easily implemented by other family medicine or internal medicine programs. The tool can and should be updated as required to remain accurate and current.

## Educational Objectives

By the end of this session participants will be able to:

1. Determine evidence-based pharmacotherapy treatment for acute and chronic conditions commonly encountered in family medicine.
2. Discuss side effects, monitoring, and clinical pearls of medications used in family medicine.

## Introduction

Pharmacotherapy is an important, required aspect of family medicine residency training. Residents must demonstrate proficiency in using pharmacotherapy. They also must utilize evidence-based medicine in practice.^1^ Therefore, an emphasis on evidence-based pharmacotherapy is valuable to incorporate into the regular education that residents receive.

Some studies have demonstrated a phenomenon known as the July effect, whereby there is an increase in medication errors with the start of a new residency year.^2,3^ A recent study addressed the rates of prescribing errors among residents.^3^ Medication classes with the highest error rates among residents included antimicrobials and anticoagulants.^3^ Likewise, rates of prescribing errors fluctuate between the residency years. Surprisingly, third-year postgraduate residents have higher error rates than second-year postgraduate residents; one would expect residents with additional experience to make fewer mistakes.^3^ Educating residents early in their residency year on the appropriate use of medications, especially those prone to medication errors, would be beneficial as a means of preventing future errors. Also, mixing the residency classes among education groups could assist them in learning from fellow residents about appropriate prescribing.

Materials have previously been created to address the deficit in family medicine education on medical errors.^4^ However, there is limited recent material that addresses the appropriate clinical use of medications. A search for *family medicine pharmacotherapy* in *MedEdPORTAL* yielded one submission from 2007.^5^ Unfortunately, information pertaining to clinical practice guidelines can become outdated as new evidence emerges. Therefore, a curriculum from 2007 has likely become outdated more than a decade later. A teaching modality that can be readily updated to reflect current practice would prove useful. A Jeopardy-style game (JSG) could serve this purpose well, since each section or question can be updated as needed. Also, JSGs are often received well and can be used in a variety of settings, with varying numbers of participants and audiences.^6–13^ This allows for significant flexibility.

A search in *MedEdPORTAL* on *family medicine pharmacology* yielded significantly more results: 53. However, they appear to be geared primarily to students, rather than residents. Adding *resident* to the search yielded 12 results. Unfortunately, the results have a relatively narrow focus. Using a JSG would allow the facilitator to cover a broader array of topics in a relatively short amount of time. Consequently, such a game could serve as a helpful tool for reviewing pharmacotherapy at the beginning or end of the resident year.

I wanted to create material that would serve as an active learning tool targeted to family medicine residents but that could be repurposed for several different audiences. Similarly, my aim was to utilize a format that would engage the audience and cover a variety of medications and guidelines. Since guidelines can change regularly, I also wanted a tool that would be easily updated. A JSG fit all these criteria.

## Methods

I created a JSG that served as a review of clinical practice guidelines and information about newly approved medications for family medicine residents. The JSG included two boards, both in Appendix A, with the second immediately following the first. The first board had the following categories: heart failure, which emphasized reduced ejection fraction heart failure; diabetes, which emphasized type 2; chronic obstructive pulmonary disease; infectious disease; and miscellaneous. The second board had the following categories: hypertension, hyperlipidemia, geriatrics 101, back pain, and anticoagulants. The JSG emphasized several current guidelines, including the American Heart Association guideline for the management of heart failure,^14^ Global Initiative for Chronic Obstructive Lung Disease,^15^ management of high blood pressure by the eighth Joint National Committee,^16^ and the guideline for management of blood cholesterol that was updated in 2018.^17^ The boards also incorporated medications likely to be approved in the future and trials that led to practice change. One or both boards could be used, depending on the amount of time available and which components needed emphasizing.

No specific preparation was required for participation by the residents. For more active participation, though, residents could be asked to review pertinent guidelines prior to the session. Likewise, the facilitator should be comfortable with the basic recommendations of the guidelines to be able to properly discuss the teaching slides and questions. At a minimum, the facilitator should review the Jeopardy boards and be acquainted with the information presented. The facilitator can also review the instructor guide (Appendix B) to assist in timing the session appropriately. Depending on the atmosphere and time frame for facilitation, residents may have further inquiries about the guidelines. Clinically relevant experience by the facilitator would be ideal to be able to respond appropriately. If the facilitator is less comfortable with specific disease states, the hidden Pertinent Guidelines references at the end of the JSG (Appendix A) can be used to find the corresponding guidelines.

I facilitated the JSG during a regularly scheduled didactic session. The residents separated into groups of three or four by residency class (i.e., one first-year postgraduate, one second-year postgraduate, etc.). The residents and I distributed Me First buzzers to each team. The buzzers allowed me to determine which team responded first; however, the use of buzzers is not necessary.^6,13^ If no technology like Me First buzzers is available, residents can simply raise their hands; the first team to have a member raise a hand would have the first opportunity to respond.^6^ As in the real Jeopardy game, the residents were expected to ask the question and were given points if they responded correctly or had points deducted if they did not. I did not enforce the standard Jeopardy rule that answers must be phrased as a question in order to collect points. At the end of the session, I gave out a hard copy of a session-specific evaluation (Appendix C) for the residents to complete. The residents also completed a standardized evaluation online that was required for attendance purposes. A sample breakdown of timing for the session is detailed in the instructor guide (Appendix B).

I created the session-specific evaluation tool (Appendix C) to determine the residents' enjoyment of the activity and their desire to have a similar session in the future. The session-specific tool expanded on the simpler standardized evaluation used for every residency didactic session. I ran only the first board for the evaluated session; due to time limitations, I could not run the second board. I also compiled the results of the session-specific evaluation tool via the Likert results and comments.

## Results

Twenty-six family medicine residents participated in the JSG during a didactic session that occurred during the first month of their residency year. Eighty-eight percent (*n* = 23) completed the session-specific evaluation; 100% completed the standardized evaluation. The results of the session-specific evaluation were positive overall. Of residents, 100% agreed or strongly agreed that the session was enjoyable, an opportunity for learning, and something they would look forward to in the future (Table). All of the residents also agreed or strongly agreed that the session provided a useful review and was a good use of time. A few residents were neutral about the difficulty of the questions, with an additional resident leaving this field blank. The appropriateness of the pace and the discussion time were the only other topics where any residents had a neutral stance. The comments mainly centered around themes of finding the information difficult or enjoying the session. Some comments contradicted each other (e.g., pace being too slow vs. requests to go slower).

• Primarily positive or neutral comments:
  ○ “Would love material for studying; trouble finding condensed sources for review.”
  ○ “Made pharm topics more interactive; made us commit to answers.”
  ○ “Overall very fun.”
  ○ “Thank you.”
  ○ “I find question and answer to be a better form to learn and retain info than lecture only.”
  ○ “The questions were very hard, but the info was very good.”
• Primarily negative comments:
  ○ “Lots of new info, but will forget.”
  ○ “[The questions] were pretty difficult.”
  ○ “Maybe make questions more specific next time.”
  ○ “Preferred questions that were more specific with one correct answer.”
  ○ “Would prefer faster pace between questions.”
  ○ “Could go slower over chronic obstructive pulmonary disease medications because many abbreviations used.”

**Table.**
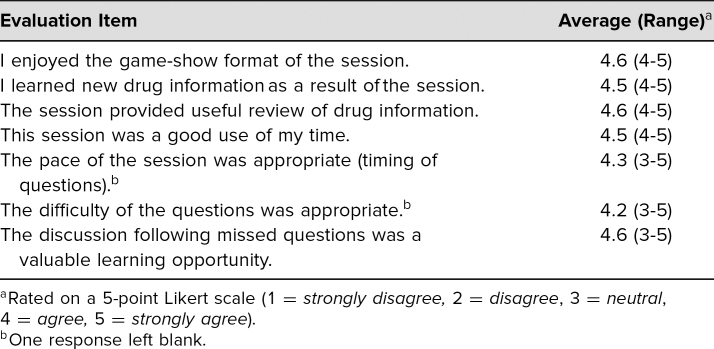
Results of Session-Specific Evaluations

The standardized questionnaire had three statements that 100% of residents agreed with: “This topic was presented in an engaging and interesting way,” “This presentation advanced my clinical and/or critical thinking skills,” and “This applied to my clinical practice.” Themes in the comments mimicked the custom evaluation, reflecting that the residents enjoyed the presentation. One resident commented, “I really liked this game and the difficulty of the questions,” which demonstrates that there was a variety of perspectives on the appropriateness of the questions.

## Discussion

To address perceived and real weaknesses in family medicine residents' pharmacotherapy knowledge, I created a JSG that would review relevant guidelines. I wanted the JSG to reflect the appropriate clinical use of medications. As had been previously discovered with other learners, the family medicine residents I taught also enjoyed and learned from a JSG.^6–12^ They expressed an interest in similar sessions in the future. Similarly, all participants felt that the information provided would apply to clinical practice, which is an important aspect of any didactic session, especially one that reviews medications. This would hopefully assist the residents in appropriate prescribing of these commonly used medications.

I also mixed the residents by residency class, which may help lead to additional education by peers. One would expect residents who are further along in their education to have more baseline knowledge of the guidelines, but this was not specifically assessed. If desired, the JSG could be updated to have a stronger emphasis on medication safety. It includes sections related to antimicrobials and anticoagulants, which are classes with high prescribing error rates by residents.^3^ It does not focus on errors that occur most frequently with the most-prescribed medications—duplicate therapy, allergies, and renal dosing.^18^ Pharmacy support and education about medication errors are both recommended components for reducing prescribing mistakes.^18^

I felt it would be helpful to create an additional evaluation because many of the sessions the residents attended did not incorporate active learning. Since it was early in the residency year, I hoped to gain additional insight into what the residents preferred. I also wanted to give future users of the JSG the opportunity to make edits based upon their more pointed feedback. The standard required evaluation contains three yes-or-no questions with one area for comments. The required evaluation serves as a tool to track attendance. Therefore, the residents complete it but may not put significant thought or effort into its completion. Based upon my previous experience, the comments often are generic (e.g., “good job”) and do not assist in making updates or changes to the presentation. I thought it would be helpful to create a feedback tool that does not fit into the typical evaluations required by family medicine residency programs and that other facilitators could use for input on their active learning. The evaluation focuses on the general resident experience rather than knowledge gained.

Based upon the results of the evaluations, it may have been better to use the JSG towards the end of the residency year. Most of the neutral evaluation results and negative comments were in relation to the difficulty of the questions, which may have been alleviated if the residents felt more comfortable with the material. This comfort with the material would more likely arise towards the end of the year, since they would have more clinical residency experience at that time. Also, I included questions about medications that were significantly less common (e.g., patiromer) or not even yet available (e.g., tecarfarin). My goal was to use the JSG to also teach new information; in hindsight, I should only have reviewed medications that were currently used in practice. Keeping up to date with current practice can be challenging enough for practicing physicians; the JSG would have likely been difficult enough without those questions. Also, I would consider removing answers that were more vague and that had multiple correct responses (e.g., nonpharmacological treatment of back pain) in future versions based upon resident feedback. Similarly, some of the questions were likely too difficult based upon the feedback and the lack of responses during the session. When I feel the questions could be removed for future iterations because they are likely too difficult, I have noted this in the JSG notes section. On the other hand, the results seem to indicate that the pace was appropriate for most participants, so following the instructor guide should be effective for most audiences.

The JSG has significant flexibility and can be used as is or updated to be more appropriate for other audiences. With very few adjustments, the JSG could be used for internal medicine residents, pharmacy students, pharmacy residents, or an interprofessional group of learners. The session would likely be too challenging for medical students. Depending on the time frame allotted and audience, questions could be collapsed into one board or skipped over.

There are some limitations of the session and evaluations used. First, the results are limited by the small sample size (*n* = 26). It would be difficult to get a larger sample size based upon the number of residents in any one program. On the other hand, the JSG could be used for residents in other specialties (e.g., internal medicine) or an interprofessional group of learners, which would allow for evaluations from a larger-sized sample. The session-specific objectives were not assessed in either evaluation used. The session-specific evaluation could have asked about the session objectives, but this would have limited its generalizability to other active learning sessions. Also, the evaluations were completed immediately after the session. Therefore, they did not assess any later changes in prescribing based upon the knowledge gained. Similarly, they did not specifically assess the knowledge gained during the session with a pre- and postquiz or test. Because of time constraints, the evaluations were based upon the facilitation of only the first JSG board. Consequently, the evaluation results do not apply directly to the full JSG. Despite these limitations, the JSG was well received overall and is a useful tool for pharmacotherapy review for family medicine residents.

## Appendices

Jeopardy-Style Pharmacotherapy Game.pptxJeopardy-Style Pharmacotherapy Game Instructor Guide.docxSession-Specific Evaluation Tool.docx All appendices are peer reviewed as integral parts of the Original Publication.
