# Supplementary material for: Pharmacotherapy Update and Review for Family Medicine Residents Using Jeopardy-Style Game
Source: MedEdPORTAL. 2020 Jul 30;16:10941. doi: 10.15766/mep_2374-8265.10941 (PMC7391450; doi:10.15766/mep_2374-8265.10941)
Supplement: Supplementary file 1 — Jeopardy-Style Pharmacotherapy Game.pptxJeopardy-Style Pharmacotherapy Game Instructor Guide.docxSession-Specific Evaluation Tool.docx [file mep_2374-8265.10941-s001.zip › B. Jeopardy-Style Pharmacotherapy Game Instructor Guide.docx]

Breakdown of session time

5-10 minutes Introduction of facilitator and rules of Jeopardy. Break up into teams and distribute buzzers

- Recommend assigning teams in advance to save time here
- Recommend groups of no more than 4 or 5, so everyone can actively engage in the group discussion

50 minutes Facilitate first board

5 minutes Break before second board

50 minutes Facilitate second board, including final jeopardy question

- Can skip to final jeopardy if running low on time

5 minutes Completion of evaluations
