# Supplementary material for: Pharmacotherapy Update and Review for Family Medicine Residents Using Jeopardy-Style Game
Source: MedEdPORTAL. 2020 Jul 30;16:10941. doi: 10.15766/mep_2374-8265.10941 (PMC7391450; doi:10.15766/mep_2374-8265.10941)
Supplement: Supplementary file 1 — Jeopardy-Style Pharmacotherapy Game.pptxJeopardy-Style Pharmacotherapy Game Instructor Guide.docxSession-Specific Evaluation Tool.docx [file mep_2374-8265.10941-s001.zip › C. Session-Specific Evaluation Tool.docx]

Please select your level of agreement with the following statements:

|  | Strongly agree | Agree | Neutral | Disagree | Strongly Disagree |
| --- | --- | --- | --- | --- | --- |
| I enjoyed the game-show format of the session. |  |  |  |  |  |
| I learned new drug information as a result of the session  **Comments:** |  |  |  |  |  |
| The session provided useful review of drug information |  |  |  |  |  |
| This session was a good use of my time |  |  |  |  |  |
| The pace of session was appropriate (timing of questions) |  |  |  |  |  |
| The difficulty of the questions was appropriate |  |  |  |  |  |
| The discussion following missed questions was a valuable learning opportunity  **Comments:** |  |  |  |  |  |
| I would look forward to more sessions like this in the future  **Comments:** |  |  |  |  |  |

**Additional comments or suggestions:**
